# Associations Between Individual‐ and Group‐Level Relational Mobility and Big Five Personality in Japan: A Multilevel Study of Prefectural Capitals

**DOI:** 10.1111/jopy.70033

**Published:** 2025-11-27

**Authors:** Takehiko Ito, Haruto Takagishi

**Affiliations:** ^1^ Hosei University Machida, Tokyo Japan; ^2^ Brain Science Institute Tamagawa University Machida, Tokyo Japan

**Keywords:** big five personality traits, group‐level, macro‐level, relational mobility

## Abstract

**Introduction:**

This study examined the associations between individual‐ and group‐level relational mobility and the Big Five personality traits, along with demographic variables and macro‐level variables, targeting 5048 people in all 47 Japanese prefectural capitals.

**Methods:**

The study controlled for macro‐level variables such as total population, population change rate, population density per 1 km^2^ of habitable area, number of incoming residents, and number of outgoing residents in each prefectural capital, which previous studies have not examined.

**Results:**

Multilevel regression analysis revealed that extraversion was positively associated with both individual‐ and group‐level relational mobility.

**Conclusion:**

The present study showed that specific features of the social environment (relational mobility) are positively associated with specific aspects of Big Five personality traits.

## 1 Introduction

Personality refers to the characteristics that describe consistent patterns of attitudes and behaviors in individuals (John et al. 2008). According to Big Five theory, personality can be categorized into five traits: extraversion, agreeableness, conscientiousness, emotional stability (reversed neuroticism), and openness (John et al. 2008). Individuals with high extraversion tend to be sociable; those with high agreeableness prefer cooperation with others; those with high conscientiousness have a strong sense of self‐discipline; those with high emotional stability (reversed neuroticism) are emotionally stable; and those with high openness are not constrained by existing values (Iwasa and Yoshida 2018). Several scales have been developed to measure these personality traits, including the Five Factor Personality Questionnaire containing 50 items (FFPQ‐50; Fujishima et al. 2005), Major Five Factor Personality Test (Murakami and Murakami 1999), Japanese versions of the NEO‐PI‐R and NEO‐FFI (Shimonaka et al. 1999), and Japanese version of the Ten‐Item Personality Inventory (TIPI‐J) (Oshio et al. 2012). The Big Five personality traits have been widely used in research to predict attitudes and behaviors.

According to Yamagishi (1998), compared to American society, interpersonal relationships and social networks in Japanese society are less flexible, where relationships are more stable and guaranteed. Consequently, individuals tend to be less proactive in interacting beyond their own groups and live in relatively closed societies. Relational mobility is the degree of freedom to choose between interpersonal relationships and social groups (Yuki et al. 2013). In other words, relational mobility is the extent to which individuals in a given society or social context have opportunities to form new interpersonal relationships as needed (Yuki et al. 2007). Relational mobility is not a personality trait or individual belief specific to a society; rather, it reflects the characteristics of the social environment, such as the networks and societal structures that surround individuals (Schug et al. 2009).

Is relational mobility related to the Big Five personality traits? Jokela (2014) found that residential mobility, which is the movement of individuals from one residential location to another, occurred among those with higher levels of openness and lower levels of emotional stability. Silventoinen et al. (2008) found that the migration from Finland to Sweden was higher among those with higher levels of extraversion and lower levels of emotional stability. Jokela (2009), targeting Americans in the 48 contiguous states with a follow‐up period of 7 to 11 years, found that higher openness was associated with higher residential mobility both within and between states. Higher extraversion was also associated with higher mobility but only for within‐state moves.

McCann (2015) found that not moving was independently associated with higher state resident levels (state's aggregate) of extraversion and lower levels of emotional stability; higher same‐county residential mobility was independently associated with lower state resident levels of extraversion and higher levels of emotional stability; and higher different‐county residential mobility was independently associated with higher state levels of emotional stability. Conscientiousness was negatively correlated with not moving but positively correlated with moving within the same county.

However, no psychological research has been conducted on the relationship between relational mobility and personality at the group level. As relational mobility reflects the characteristics of the social environment, such as the networks and societal structures that surround individuals (Schug et al. 2009), a macro‐level analysis of relational mobility is necessary to examine the association between the environment and personality. This study is important because it is the first to explore the aggregate personality differences between people's relational mobility in 47 prefectures in Japan, which can account for the variance in personal tendencies. This study focused on the association between group‐level relationship mobility and group‐level personality, and the relationship between individual‐level relationship mobility and individual‐level personality. Some differences between areas can be understood as aggregate outcomes of the corresponding dispositional variables at the individual level, assuming that the hypotheses at the individual level will be applicable in the context of aggregate‐level relations (McCann 2015). We applied psychological theories and research findings to aggregate‐level relations using a multilevel analysis. Multilevel analysis is a methodology used to test how individual‐level findings manifest at the aggregate level. The following hypotheses regarding the Big Five personality traits are predictions based on individual‐level findings:

Individuals in highly relationally mobile environments, such as those in Western societies, are more motivated to develop and strengthen new relationships than individuals in relationally stable environments (Schug et al. 2010). In contrast, individuals in relationally stable cultures, such as Singapore and Taiwan, have fewer opportunities to meet new people and less freedom to form or dissolve relationships at will (Wang et al. 2011). Additionally, a comparison between Asian and European Canadians found that European Canadians exhibited higher relational mobility and were more likely to engage in self‐disclosure with close friends than Asian Canadians (Zhang and Li 2014). Those higher on extraversion have qualities such as sociability and talkativeness that make it easier to settle into a new milieu (McCann 2015). These findings suggest that a relationally mobile environment is associated with proactive engagement with new people and the tendency to express oneself to friends. Therefore, relational mobility is hypothesized to be positively associated with extraversion at both individual and group levels.

What about agreeableness? A comparison between Japanese and American individuals found that Americans, who experience higher relational mobility, are more likely to perceive their friends as similar to themselves (Schug et al. 2009). Similarly, a study comparing Asian and European Canadians revealed that European Canadians exhibit higher relational mobility, which is linked to a greater tendency to perceive close friends as similar (Zhang and Li 2014). Agreeableness has been reported to be negatively correlated with the perception of dissimilarity between oneself and others (Liao et al. 2008). This suggests that a relationally mobile environment fosters a greater sense of similarity with others and a stronger tendency toward cooperation. Agreeable people show higher trust and generosity and tend to be better liked than disagreeable people, which could result in stronger ties to their present community or make it easier to adjust to a new one (McCann 2015). Therefore, relational mobility is hypothesized to be positively associated with agreeableness at both individual and group levels.

What about emotional stability? A study comparing Japanese and Canadian individuals found that Canadians, who experience higher relational mobility, have higher self‐evaluation, which is linked to their level of relational mobility (Falk et al. 2009). Similarly, a study comparing Asian and European Canadians confirmed that European Canadians exhibited higher relational mobility and self‐esteem (Zhang and Li 2014). Notably, emotional stability is positively correlated with self‐esteem (Nozaki and Koyasu 2015). Additionally, research has shown that Japanese individuals experience lower relational mobility than British and American individuals, and are more likely to feel shame in front of friends (Sznycer et al. 2012). Furthermore, European Canadian residents exhibit higher relational mobility and lower rejection sensitivity than Hong Kong residents (Lou and Li 2017). These findings suggest that relational mobility environments are associated with greater emotional stability and lower sensitivity toward others. Therefore, relational mobility is hypothesized to be positively associated with emotional stability at both individual and group levels.

Regarding openness, a study comparing European Canadian residents and Hong Kong residents found that European Canadians, who exhibited higher relational mobility, were more likely to endorse a growth mindset—the belief that relationships improve through effort—while being less likely to endorse a fate‐based mindset, which assumes that relationship success is predetermined (Lou and Li 2017). This suggests that a relational mobility environment is associated with open‐minded growth‐oriented thinking rather than fixed fate‐driven beliefs. Those higher in openness show greater curiosity and willingness to experiment, and openness may predispose a person to move, perhaps to a very different location (McCann 2015). Therefore, relational mobility is hypothesized to be positively associated with openness at both individual and group levels.

Regarding conscientiousness, the greater sense of organization, self‐discipline, responsibility, and persistence in those with higher conscientiousness could motivate them to move or stay in one place, depending on situational factors such as family and career pressures (McCann 2015). Therefore, due to the offset, relational mobility is hypothesized to show no associations with conscientiousness.

In the present study, individuals were nested within prefectures (Nezlek 2008). The measured factors are typically calculated based on individuals as independent factors. However, in this situation, each prefecture had its own intra‐class correlation of relational mobility. As the assumption of a parametric statistical test is the independence of individual observations, this phenomenon violates this assumption (Hox 2010). Therefore, a multilevel analysis was employed to address this problem. This method treats individuals and groups hierarchically and simultaneously estimates each individual's fixed effects and each group's random effects (Shimizu 2014).

The hypotheses of the present study were tested at level 1 (individual) using the following model:

$$Y_{\mathrm{ij}}=\beta_{0j}+{\beta_{1j}}^{*}\;{\text{Relational Mobility}}_{\mathrm{ij}}+e_{\mathrm{ij}}$$

The hypotheses of this study were also tested at Level 2 (prefecture) using the following model:

$$\beta_{0j}=\gamma_{00}+{\gamma_{01}}^{*}\;\text{Prefecture}-\text{Level Relational Mobility}+u_{0j}$$

$$\beta_{1j}=\gamma_{10}+{\gamma_{11}}^{*}\;\text{Prefecture}-\text{Level Relational Mobility}+u_{1j}$$

where *Y*_*ij*_ indicates the Big Five personality traits score for member *i* in prefecture *j*, *β*_0*j*_ indicates the intercept, and *β*_1*j*_ is the regression coefficient. *β*_0*j*_ and *β*_1*j*_ were estimated using *γ*_00_ and *γ*_10_ (fixed effects), and *u*_0*j*_ and *u*_1j_ (random effects), respectively. The formulas for the control variables are abbreviated in these models.

## 2 Methods

### 2.1 Participants

The participants were 5048 Japanese individuals (2519 men, 2529 women; mean age = 31.32 years, SD = 5.52) living in the 47 prefectural capitals (all prefectures of Japan). We collected data from participants in their twenties and thirties. Relational mobility may differ among cities, even within the same prefecture. Therefore, this study targeted people living in each of the prefectural capitals (typical cities) to limit the variability of relational mobility within each prefecture. According to the G*Power 3.1.9.7 software (Faul et al. 2009), at 0.95 power in multiple linear regression, a minimum sample of 89 individuals was necessary. The subsample size of the 47 capital cities was approximately 100. For multilevel analysis, we required a valid sample for each group. Therefore, the sample size of the survey was sufficient.

### 2.2 Procedure

A web‐based survey tool was used to collect the data (collected on March 16, 2022). Macromill sent links to the online survey to the participants who could then access the links on their personal computers or smartphones. The first page indicated that participation was voluntary and anonymous. The participants provided informed consent to participate and received compensation. The survey company removed invalid participants (e.g., those who answered the questionnaires unreliably before) before sending them the survey link. We therefore retain standard exclusions.

For demographic variables, sex, age, number of moves, number of new people met in 1 month, and number of new people met in 3 months were recorded. Macro‐level variables, such as total population, population change rate, population density per 1 km^2^ of habitable area, number of incoming residents, and number of outgoing residents in each prefectural capital, were extracted from the 2020 Census of Japan.

The studies involving human participants were reviewed and approved by the Research Committee of the Department of Information Networking for Innovation and Design at Toyo University, Japan.

### 2.3 Questionnaires

#### 2.3.1 Relational Mobility

Yuki et al. (2007) developed the Relational Mobility Scale and used its Japanese version in this study. The participants answered items relating to how many statements accurately described people in their immediate society (school, workplace, town, neighborhood, etc.). The scale included two dimensions: items related to meeting new people (e.g., “They have many chances to get to know other people,” “It is common for these people to have a conversation with someone they have never met before”) and items related to the choice of one's own interaction partners (e.g., “They can choose who they interact with,” “If they did not like their current groups, they would leave for better ones”). Twelve items were used to measure relational mobility (*α* = 0.66). Response options ranged from 1 (*not at all*) to 7 (*very much*).

#### 2.3.2 Big Five Personality Traits

The Japanese version (Oshio et al. 2012) of the short form of the Big‐Five scale, the “Ten Item Personality Inventory” (Gosling et al. 2003), was used; its reliability and validity have been confirmed (Namikawa et al. 2012). Participants were asked to indicate the extent of their perceived personality traits by responding to ten items. This scale consisted of five personality factors: extraversion: “extraverted, enthusiastic” and “reserved, quiet” (reverse‐scored; *r* = 0.33, *p <* 0.01); agreeableness: “sympathetic, warm” and “critical, quarrelsome” (reverse‐scored; *r* = 0.18, *p <* 0.01); conscientiousness: “dependable, self‐disciplined” and “disorganized, careless” (reverse‐scored; *r* = 0.28, *p <* 0.01); emotional stability: “anxious, easily upset” (reverse‐scored) and “calm, emotionally stable” (*r* = 0.25, *p <* 0.01); and openness: “open to new experiences, complex” and “conventional, uncreative” (reverse‐scored; *r* = 0.21, *p <* 0.01). The response options for all the statements ranged from 1 (*strongly disagree*) to 7 (*strongly agree*).

#### 2.3.3 Number of Moves

Number of moves was measured using a 6‐point Likert scale ranging from 1 (never) to 6 (over five times). The question is “How many times in total have you moved to a new area or a different municipality since the age of five?” If you moved before the age of five or only moved within the same area or municipality, select “1 (never).”

#### 2.3.4 Number of New People Met in 1 Month

Number of new people met in 1 month was assessed using the open‐ended question “How many new people have you met in the past month? Please provide an approximate number.”

#### 2.3.5 Number of New People Met in 3 Months

The number of new people met in 3 months was measured using the open‐ended question “How many new people have you met in the past three months? Please provide an approximate number.”

#### 2.3.6 Total Population in Each Prefectural Capital

The total population of each prefectural capital was extracted from the Census of Japan 2020.

#### 2.3.7 Population Change Rate in Each Prefectural Capital

The population change rate in each prefectural capital was extracted from the Census of Japan 2020. The rate of population change was calculated by dividing the net change in total population from October of the previous year to September of the current year by the population at the beginning of the period.

#### 2.3.8 Population Density Per 1 km^2^ of Habitable Area in Each Prefectural Capital

Population density per 1 km^2^ of habitable area was extracted from the Census of Japan 2020. Population density per 1 km^2^ of habitable area was calculated by dividing the population of habitable land by the area of habitable land.

#### 2.3.9 Number of Incoming Residents in Each Prefectural Capital

The number of incoming residents was extracted from the Census of Japan 2020.

#### 2.3.10 Number of Outgoing Residents in Each Prefectural Capital

The number of outgoing residents was extracted from the Census of Japan 2020.

## 3 Results

The means, standard deviations, and correlation matrices are presented in Table 1. The extraversion (*M* = 3.50, SD = 1.29), agreeableness (*M* = 4.55, SD = 1.09), conscientiousness (*M* = 3.64, SD = 1.15), emotional stability (*M* = 3.57, SD = 1.15), openness (*M* = 3.72, SD = 1.14), individual‐level relational mobility (*M* = 4.12, SD = 0.58), and group‐level relational mobility scores (*M* = 4.12, SD = 0.07) were around the midpoint.

**TABLE 1:** Means, standard deviations, and correlations matrix.

	*M*	SD	1	2	3	4	5	6	7	8	9	10	11	12	13	14	15	16
1. Extraversion	3.50	1.29	—															
2. Agreeableness	4.55	1.09	−0.08**	—														
3. Conscientiousness	3.64	1.15	0.24**	0.16**	—													
4. Emotional stability	3.57	1.15	0.27**	0.17**	0.32**	—												
5. Openness	3.72	1.14	0.34**	0.01	0.23**	0.25**	—											
6. Relational mobility_Individual	4.12	0.58	0.11**	0.10**	0.02	0.04**	0.06**	—										
7. Relational mobility_Group	4.12	0.07	0.05**	0.02	0.03*	0.03*	0.02	0.13**	—									
8. Sex (*M* = 1, *W* = 2)	1.50	0.50	0.02	0.08**	−0.04**	−0.19**	−0.13**	0.08**	0.00	—								
9. Age	31.32	5.52	−0.02	−0.02	0.05**	0.01	−0.05**	−0.08**	−0.02	−0.08**	—							
10. Number of moves	3.09	1.66	0.06**	0.06**	0.05**	0.05**	0.02+	0.00	0.03*	0.00	0.22**	—						
11.1 Month_New people met	2.54	17.41	0.05**	0.00	0.00	0.03*	0.04**	0.03*	0.02	−0.02^+^	−0.05**	0.00	—					
12.3 Months_New people met	7.37	102.50	0.03*	−0.01	0.01	0.03*	0.02	0.02+	0.01	−0.02^+^	−0.02	0.00	0.74**	—				
13. Total population	932,564.02	1,516,652.81	0.04*	0.00	0.02+	0.04**	0.02	0.02	0.13**	0.00	0.00	0.03*	0.00	−0.01	—			
14. Population change rate	−0.66	2.10	0.03*	0.00	0.03*	0.03*	0.01	0.03*	0.25**	0.00	0.00	0.06**	0.00	0.00	0.63**	—		
15. Population density per 1 km^2^	3225.66	3121.05	0.04**	0.00	0.03+	0.04**	0.02	0.03+	0.21**	0.00	0.01	0.03*	−0.01	−0.01	0.85**	0.71**	—	
16. Number of incoming residents	49,703.46	105,504.02	0.04**	0.00	0.02+	0.04**	0.02	0.02	0.13**	0.00	0.00	0.03*	0.00	−0.01	1.00**	0.63**	0.85**	—
17. Number of outgoing residents	48,102.07	102,523.66	0.04**	0.00	0.02+	0.04**	0.02	0.02	0.14**	0.00	0.00	0.03*	0.00	−0.01	1.00**	0.62**	0.84**	1.00**

** *p* < 0.01.

* *p* < 0.05.

^+^ *p* < 0.10.

Individual‐level relational mobility was positively correlated with extraversion (*r* = 0.11, *p* < 0.01), agreeableness (*r* = 0.10, *p* < 0.01), emotional stability (*r* = 0.04, *p* < 0.01), and openness (*r* = 0.06, *p* < 0.01) but not with conscientiousness (*r* = 0.02, n.s. (not significant)). Group‐level relational mobility was positively correlated with extraversion (*r* = 0.05, *p* < 0.01), conscientiousness (*r* = 0.03, *p* < 0.05), and emotional stability (*r* = 0.03, *p* < 0.05) but not with agreeableness (*r* = 0.02, n.s.) and openness (*r* = 0.02, n.s.).

Regarding macro‐level variables, individual‐level relational mobility was positively correlated with the population change rate in each prefectural capital (*r* = 0.03, *p* < 0.05) and slightly correlated with population density per 1 km^2^ of habitable area in each prefectural capital (*r* = 0.03, *p* < 0.10). Group‐level relational mobility was positively correlated with all the macro‐level variables. Furthermore, extraversion, conscientiousness, and emotional stability were positively correlated with all macro‐level variables, whereas agreeableness and openness were not.

First, without any control variables, the association between individual‐ and group‐level relational mobility and the Big Five personality traits was analyzed (Table 2). Multilevel regression analysis showed that extraversion was positively related to both individual‐ and group‐level relational mobility (*β* = 0.23, SE = 0.03, 95% CI [0.16, 0.30], *p* < 0.01; *β* = 0.91, SE = 0.26, 95% CI [0.38, 1.44], *p* < 0.01). For agreeableness, it was positively related to individual‐level relational mobility (*β* = 0.18, SE = 0.03, 95% CI [0.13, 0.23], *p* < 0.01), but not to group‐level relational mobility (*β* = 0.33, SE = 0.21, 95% CI [−0.08, 0.75], n.s.). For conscientiousness, it was positively related to group‐level relational mobility (*β* = 0.52, SE = 0.23, 95% CI [0.06, 0.98], *p* < 0.05), but not to individual‐level relational mobility (*β* = 0.04, SE = 0.03, 95% CI [−0.02, 0.09], n.s.). For emotional stability, it was positively related to both individual‐ and group‐level relational mobility (*β* = 0.08, SE = 0.03, 95% CI [0.02, 0.13], *p* < 0.01; *β* = 0.47, SE = 0.22, 95% CI [0.03, 0.91], *p* < 0.05). For openness, it was positively related to individual‐level relational mobility (*β* = 0.12, SE = 0.04, 95% CI [0.04, 0.19], *p* < 0.01), but not to group‐level relational mobility (*β* = 0.27, SE = 0.22, 95% CI [−0.17, 0.72], n.s.).

**TABLE 2:** Association between individual‐ and group‐level relational mobility and the Big Five personality traits.

	Extraversion	Agreeableness	Conscientiousness	Emotional stability	Openness
Relational mobility_Individual	0.23**	0.18**	0.04	0.08**	0.12**
Relational mobility_Group	0.91**	0.33	0.52*	0.47*	0.27

** *p* < 0.01, standardized coefficient.

* *p* < 0.05, standardized coefficient.

Next, for demographic variables such as sex, age, number of moves, number of new people met in one and 3 months, the association between individual‐ and group‐level relational mobility and the Big Five personality traits was analyzed (Table 3). Multilevel regression analysis showed that extraversion was positively related to both individual‐ and group‐level relational mobility (*β* = 0.22, SE = 0.03, 95% CI [0.15, 0.29], *p* < 0.01; *β* = 0.91, SE = 0.26, 95% CI [0.39, 1.44], *p* < 0.01). For agreeableness, it was positively related to individual‐level relational mobility (*β* = 0.17, SE = 0.03, 95% CI [0.11, 0.22], *p* < 0.01), but not to group‐level relational mobility (*β* = 0.34, SE = 0.20, 95% CI [−0.07, 0.75], n.s.). For conscientiousness, it was positively related to group‐level relational mobility (*β* = 0.52, SE = 0.23, 95% CI [0.06, 0.98], *p* < 0.05), but not to individual‐level relational mobility (*β* = 0.05, SE = 0.03, 95% CI [−0.01, 0.11], n.s.). For emotional stability, it was positively related to both individual‐ and group‐level relational mobility (*β* = 0.10, SE = 0.03, 95% CI [0.05, 0.16], *p* < 0.01; *β* = 0.47, SE = 0.21, 95% CI [0.04, 0.90], *p* < 0.05). For openness, it was positively related to individual‐level relational mobility (*β* = 0.12, SE = 0.04, 95% CI [0.05, 0.20], *p* < 0.01), but not to group‐level relational mobility (*β* = 0.28, SE = 0.22, 95% CI [−0.17, 0.72], n.s.). These results were not different from the previous results (Table 2).

**TABLE 3:** Association between individual‐ and group‐level relational mobility and the Big Five personality traits after controlling for demographic variables.

	Extraversion	Agreeableness	Conscientiousness	Emotional stability	Openness
Relational mobility_Individual	0.22**	0.17**	0.05	0.10**	0.12**
Relational mobility_Group	0.91**	0.34	0.52*	0.47*	0.28
Sex (*M* = 1, *W* = 2)	0.02	0.15**	−0.09**	−0.44**	−0.31**
Age	−0.01	0.00	0.01**	0.00	−0.01**
Number of moves	0.05**	0.04**	0.03**	0.03**	0.02*
1 Month_New people met	0.00**	0.00	0.00	0.00	0.00*
3 Months_New people met	0.00	0.00	0.00	0.00	0.00

** *p* < 0.01, standardized coefficient.

* *p* < 0.05, standardized coefficient.

Finally, using demographic variables and macro‐level variables such as total population, population change rate, population density per 1 km^2^ of habitable area, number of incoming residents, and number of outgoing residents in each prefectural capital, the association between individual‐ and group‐level relational mobility and the Big Five personality traits was analyzed (Table 4). Multilevel regression analysis showed that extraversion was positively related to both individual‐ and group‐level relational mobility (*β* = 0.22, SE = 0.03, 95% CI [0.15, 0.29], *p* < 0.01; *β* = 0.62, SE = 0.28, 95% CI [0.07, 1.18], *p* < 0.05). For agreeableness, it was positively related to individual‐level relational mobility (*β* = 0.17, SE = 0.03, 95% CI [0.11, 0.22], *p* < 0.01), but not to group‐level relational mobility (*β* = 0.35, SE = 0.23, 95% CI [−0.12, 0.81], n.s.). For conscientiousness, it was not related to either individual‐ or group‐level relational mobility (*β* = 0.05, SE = 0.03, 95% CI [−0.01, 0.11], n.s.; *β* = 0.28, SE = 0.25, 95% CI [−0.23, 0.78], n.s.). For emotional stability, it was positively related to individual‐level relational mobility (*β* = 0.10, SE = 0.03, 95% CI [0.05, 0.16], *p* < 0.01), but not to group‐level relational mobility (*β* = 0.27, SE = 0.24, 95% CI [−0.22, 0.75], n.s.). For openness, it was positively related to individual‐level relational mobility (*β* = 0.12, SE = 0.04, 95% CI [0.05, 0.20], *p* < 0.01), but not to group‐level relational mobility (*β* = 0.18, SE = 0.24, 95% CI [−0.31, 0.67], n.s.). In contrast to the results in Table 3, the group‐level association between relational mobility and conscientiousness and emotional stability disappeared after controlling for macro‐level variables.

**TABLE 4:** Association between individual‐ and group‐level relational mobility and the Big Five personality traits after controlling for demographic and macro‐level variables.

	Extraversion	Agreeableness	Conscientiousness	Emotional stability	Openness
Relational mobility_Individual	0.22**	0.17**	0.05	0.10**	0.12**
Relational mobility_Group	0.62*	0.35	0.28	0.27	0.18
Sex (*M* = 1, *W* = 2)	0.02	0.15**	−0.09**	−0.44**	−0.31**
Age	−0.01	0.00	0.01**	0.00	−0.01**
Number of moves	0.05**	0.04**	0.03**	0.03**	0.02*
1 Month_New people met	0.00**	0.00	0.00	0.00	0.00*
3 Months_New people met	0.00	0.00	0.00	0.00	0.00
Total population	0.00	0.00	0.00	0.00	0.00
Population change rate	0.01	0.00	0.02	0.00	0.00
Population density per 1 km^2^	0.00	0.00	0.00	0.00	0.00
Number of incoming residents	0.00^+^	0.00	0.00	0.00	0.00
Number of outgoing residents	0.00^+^	0.00	0.00	0.00	0.00

** *p* < 0.01, standardized coefficient.

* *p* < 0.05, standardized coefficient.

^+^ *p* < 0.10, standardized coefficient.

## 4 Discussion

The results of the correlational analysis showed that individual‐level relational mobility was positively correlated with extraversion, agreeableness, emotional stability, and openness, but not with conscientiousness. Group‐level relational mobility was positively correlated with extraversion, conscientiousness, and emotional stability, but not with agreeableness and openness.

Regarding the macro‐level variables, individual‐level relational mobility was positively correlated with the population change rate in each prefectural capital and slightly correlated with population density per 1 km^2^ of habitable area in each prefectural capital. Group‐level relational mobility was positively correlated with all the macro‐level variables. Furthermore, extraversion, conscientiousness, and emotional stability were positively correlated with all macro‐level variables, whereas agreeableness and openness were not.

For extraversion, by examining the association between individual‐ and group‐level relational mobility and the trait without any control variables, a multilevel regression analysis showed a positive association between individual‐ and group‐level relational mobility and extraversion. With respect to demographic variables, the multilevel regression analysis showed the same tendency. Furthermore, with the demographic and macro‐level variables, the multilevel regression analysis showed the same tendency. Thus, the hypothesis that relational mobility was positively associated with extraversion at both individual and group levels was confirmed. As stated above, those higher in extraversion have qualities such as sociability and talkativeness, which make it easier for them to settle into a new milieu (McCann 2015). Based on previous research, it has been assumed that a highly relationally mobile environment is associated with proactive engagement with new people and the tendency to express oneself to friends.

For agreeableness, by examining the association between individual‐ and group‐level relational mobility and traits without any control variables, a multilevel regression analysis showed that individual‐level relational mobility was positively related to agreeableness, but group‐level relational mobility was not. With respect to demographic variables, multilevel regression analysis showed the same tendency. With demographic and macro‐level variables, the multilevel regression analysis showed the same tendency. Thus, the hypothesis that relational mobility was positively associated with agreeableness at both individual and group levels was not confirmed. As stated above, agreeable people show higher trust and generosity, and tend to be better liked than disagreeable people, which could result in stronger ties to their present community or make it easier to adjust to a new one (McCann 2015). In the present study, agreeableness meant stronger ties to the community, which did not motivate them to move outside of their community. Therefore, group‐level relational mobility was not related to agreeableness.

For emotional stability, by examining the association between individual‐ and group‐level relational mobility and the trait without any control variables, a multilevel regression analysis showed the positive association between individual‐ and group‐level relational mobility and emotional stability. With respect to demographic variables, multilevel regression analysis showed the same tendency. However, with demographic and macro‐level variables, the multilevel regression analysis showed that individual‐level relational mobility was positively associated with emotional stability, but group‐level relational mobility was not. Thus, the hypothesis that relational mobility was positively associated with emotional stability at both individual and group levels was not confirmed. As stated above, previous research has shown that Japanese individuals experience lower relational mobility than British and American individuals and are more likely to feel shame in front of friends (Sznycer et al. 2012), and a relationally mobile environment is associated with greater emotional stability and lower sensitivity to others. However, emotional stability in the present study meant stronger ties to the community, where they felt relaxed and emotionally stable. Therefore, group‐level relational mobility was not related to emotional stability.

For openness, by examining the association between individual‐ and group‐level relational mobility and the trait without any control variables, a multilevel regression analysis showed that individual‐level relational mobility was positively related to openness but group‐level relational mobility was not. With respect to demographic variables, the multilevel regression analysis showed the same tendency. With demographic and macro‐level variables, the multilevel regression analysis showed the same tendency. Thus, the hypothesis that relational mobility was positively associated with openness at both individual and group levels was not confirmed. As stated above, a study comparing European Canadians and Hong Kong residents found that European Canadians, who exhibited higher relational mobility, were more likely to endorse a growth mindset—the belief that relationships improve through effort—while being less likely to endorse a fate‐based mindset, which assumes that relationship success is predetermined (Lou and Li 2017). However, in the present study, openness meant stronger ties to the community, where they could have had a more open‐minded, growth‐oriented belief system. Therefore, group‐level relational mobility was not related to openness.

For conscientiousness, by examining the association between individual‐ and group‐level relational mobility and the trait without any control variables, a multilevel regression analysis showed that group‐level relational mobility was positively related to conscientiousness, but individual‐level relational mobility was not. With respect to the demographic variables, the multilevel regression analysis showed the same tendency. With demographic and macro‐level variables, the multilevel regression analysis showed no association between individual‐ or group‐level relational mobility and conscientiousness. As a hypothesis, relational mobility showed no associations with conscientiousness, which was confirmed. As stated above, a greater sense of organization, self‐discipline, responsibility, and persistent conscientiousness could motivate one to move or stay in one place, depending on situational factors, such as family and career pressures (McCann 2015). Therefore, due to the offset, relational mobility was not related to conscientiousness.

People in East Asia do not have many opportunities to meet new people or terminate interpersonal relationships; this is referred to as lower relational mobility (Wang et al. 2011). By contrast, people in Europe and America with higher relational mobility have such opportunities and create new interpersonal relationships (Schug et al. 2010). To generalize the results of the present study, it is necessary to examine the macro‐level association between relational mobility and the Big Five personality traits by targeting countries with high relational mobility.

## 5 Limitation

Prefectural capitals in Japan are not necessarily the largest cities in their respective regions, nor are they always the cities with the longest history. Some prefectures have cities larger than their capitals, while others have cities comparable in size to their capitals. Future work should consider sampling multiple municipalities per prefecture.

We acknowledge that our interpretation of the non‐significant associations as reflecting stronger ties to the community is post hoc and should therefore be considered exploratory. Future research could further examine the relationship between relational mobility and personality traits using measures that directly operationalize community embeddedness.

## 6 Conclusion

This study showed the association between individual‐ and group‐level relational mobility and the Big Five personality traits with and without demographic variables, targeting people in all 47 Japanese prefectural capitals. Furthermore, the study controlled for macro‐level variables such as total population, population change rate, population density per 1 km^2^ of habitable area, number of incoming residents, and number of outgoing residents in each prefectural capital, which previous studies have not examined. Multilevel regression analysis revealed that extraversion was positively related to individual‐ and group‐level relational mobility. The present study showed that specific features of the social environment (relational mobility) are positively associated with specific aspects of Big Five personality traits.

## Author Contributions

**Takehiko Ito:** conceptualization, methodology, formal analysis; Data curation, writing – original draft; visualization, Funding acquisition. **Haruto Takagishi:** conceptualization, methodology, resources, supervision, Funding acquisition, writing – review and editing.

## Funding

This study was supported by the Inoue Enryo Memorial Grant 2021, Toyo University, Toyo University and JSPS KAKENHI (Grant JP24H00910).

## Conflicts of Interest

The authors declare no conflicts of interest.
